# Clinical Trials Using Mesenchymal Stem Cells for Spinal Cord Injury: Challenges in Generating Evidence

**DOI:** 10.3390/cells11061019

**Published:** 2022-03-17

**Authors:** Lila Teixeira de Araújo, Carolina Thé Macêdo, Patrícia Kauanna Fonseca Damasceno, Ítalo Gabriel Costa das Neves, Carla Souza de Lima, Girlaine Café Santos, Thaís Alves de Santana, Gabriela Louise de Almeida Sampaio, Daniela Nascimento Silva, Cristiane Flora Villarreal, Alessandra Casemiro de Campos Chaguri, Crislaine Gomes da Silva, Augusto César de Andrade Mota, Roberto Badaró, Ricardo Ribeiro dos Santos, Milena Botelho Pereira Soares

**Affiliations:** 1 Institute of Advanced Systems in Health, Senai Cimatec, Salvador 41650-010, BA, Brazil; lila.araujo@fieb.org.br (L.T.d.A.); carolthemacedo@gmail.com (C.T.M.); patricia.damasceno@fieb.org.br (P.K.F.D.); italo.neves@ufba.br (Í.G.C.d.N.); carlalimafisioterapia@gmail.com (C.S.d.L.); girlainecafe@gmail.com (G.C.S.); thaisantana669@gmail.com (T.A.d.S.); gabrielalouise.sampaio@gmail.com (G.L.d.A.S.); daniela.silva@ki.se (D.N.S.); alessandrachaguri@gmail.com (A.C.d.C.C.); crisgdasilva22@gmail.com (C.G.d.S.); badaro@fieb.org.br (R.B.); ricardoribeiro1941@gmail.com (R.R.d.S.); 2 Gonçalo Moniz Institute, Oswaldo Cruz Foundation (FIOCRUZ), Salvador 40296-710, BA, Brazil; cfv@ufba.br; 3 Climério de Oliveira Hospital, Federal University of Bahia, Salvador 40055-150, BA, Brazil; 4 Karolinska Institutet, Department of Laboratory Medicine, 14152 Stockolm, Sweden; 5 Faculty of Pharmacy, Federal University of Bahia, Salvador 40170-115, BA, Brazil; 6 AMO Clinic/Etica Institute, Salvador 41950-640, BA, Brazil; augustomota@clinicaamo.com.br

**Keywords:** spinal cord injury, mesenchymal stem cells, mesenchymal stromal cells, clinical trial

## Abstract

Spinal cord injury (SCI) remains an important public health problem which often causes permanent loss of muscle strength, sensation, and function below the site of the injury, generating physical, psychological, and social impacts throughout the lives of the affected individuals, since there are no effective treatments available. The use of stem cells has been investigated as a therapeutic approach for the treatment of SCI. Although a significant number of studies have been conducted in pre-clinical and clinical settings, so far there is no established cell therapy for the treatment of SCI. One aspect that makes it difficult to evaluate the efficacy is the heterogeneity of experimental designs in the clinical trials that have been published. Cell transplantation methods vary widely among the trials, and there are still no standardized protocols or recommendations for the therapeutic use of stem cells in SCI. Among the different cell types, mesenchymal stem/stromal cells (MSCs) are the most frequently tested in clinical trials for SCI treatment. This study reviews the clinical applications of MSCs for SCI, focusing on the critical analysis of 17 clinical trials published thus far, with emphasis on their design and quality. Moreover, it highlights the need for more evidence-based studies designed as randomized controlled trials and potential challenges to be addressed in context of stem cell therapies for SCI.

## 1. Introduction

Traumatic spinal cord injury (SCI) is a relatively common medical problem, with incidence ranging from 10 to 80 cases per million people each year [1,2,3]. SCI may cause motor and sensory dysfunction, leading to a diverse array of sequelae depending on the primary level of the injury, and ultimately, will compromise the individual’s performance in carrying out activities of daily living and social interaction. The affected individuals also have a two to three times higher incidence of mental disorders when compared to the general population, which usually are not addressed due to the large impacts of the motor and sensory impairments [4]. Moreover, the decreases in productivity and quality of life of these patients have a considerable negative impact on the economy of both families and society [5]. Finally, costs of SCI are estimated to be 10 billion dollars a year, varying according to phase, level, and severity of the injury [1,6]. 

The potentially permanent and usually progressive consequences of SCI are due to a set of anatomopathological events that occur in the nervous tissue after the trauma, which results in hemorrhages, edema, and tissue death due to necrosis at the injury site. The combination of a traumatic impact with compression of bone and other parts of the spine against the spinal cord can also promote ischemia that increases the tissue damage [7,8]. Moreover, an inflammatory process results due to the trauma, causing processes such as apoptosis of different cell populations (including neurons, astrocytes, and oligodendrocytes), a reduction in the transmission of nerve impulses, and an increase in demyelination [9,10,11]. Furthermore, other processes may contribute to the impairment of neurological function and reduced tissue regeneration [12,13], such as activation of resident microglia, which increases in tissue damage [14]; formation of gliosis and glial scarring, which impairs nerve impulse conduction; and decreased neural growth [15].

Currently, the therapeutic options available for restoring the neurological functions lost after SCI include pharmacotherapy, physical therapy, and surgical decompression, all showing low effectiveness and short-lasting improvements [16,17,18,19,20,21]. In view of these limited treatment options, new therapeutic approaches for the management of SCI are needed. The development of cell-based therapies has been considered an innovative approach which may bring more significant and long-lasting improvements in sensory and motor outcomes [22,23,24].

Several cell types and sources have been tested in animal models of SCI, including neural stem cells, embryonic stem cells, induced pluripotent stem cells, and mesenchymal stem/stromal cells (MSCs). The cell type most commonly tested is MSCs, which can be easily obtained from different sources, such as bone marrow, adipose tissue, and Wharton’s jelly from the umbilical cord. In attempts to show the safety and efficacy of this therapy in SCI, several preclinical studies using MSCs have been carried out, showing promising results [25,26,27,28,29,30]. MSCs are an attractive cell type, widely shown to be safe and potentially beneficial in several disease settings, including neurological diseases. The findings reported in animal SCI models showed that transplanted MSCs migrate to regions of experimentally induced nerve damage, where they can proliferate and differentiate into neurons and glial cells, leading to neuroprotective and neuroregenerative effects through prevention of cell death, and an increase in tissue integrity in the injured segment of the spinal cord [31,32]. This may ultimately result in the restoration of motor and sensory functions in SCI models, acting through the modulation of inflammation, axonal preservation, glial remodeling, increased angiogenesis, and reducing the formation of cavity lesions [33,34].

In recent years, several studies have been published trying to elucidate the mechanisms by which MSCs play their roles as tissue homeostasis maintainers and as tools for regenerative medicine. Despite the evidence on the direct differentiation and cell replacement of MSCs, most reports strongly suggest that the most notable mechanisms of action of MSCs are their ability to exert paracrine effects [35,36] and their modulatory action on the immune system (immunomodulation) [37,38]. Furthermore, MSCs are able to promote trophic and regenerative support to injured tissues, mainly through the secretion of soluble factors, such as cytokines and growth factors; release of extracellular vesicles containing proteins, messenger RNA (mRNA), and microRNAs; and mitochondrial transfer, thereby promoting autocrine and paracrine effects, such as immunosuppression, inhibition of gliosis and apoptosis, increased angiogenesis, axonal growth, myelination, and neuronal survival (for damaged neurons) [39,40,41,42].

Despite the advances in animal models, data from human trials have been less promising. Beyond the anatomic and intrinsic differences between the spontaneous regeneration of mouse and human spinal cords that are already known, in animal studies researchers are able to standardize protocols and choose similar models that present better responses for spinal cord injuries. These conditions are more difficult to achieve in trials involving humans due to different and unexpected variables that need to be controlled to obtain reliable outcomes.

There are currently no recommendations from international committees and societies, nor there are standardized protocols that would assure the safety and efficacy related to the use of MSC therapy for the treatment of SCI, and there is no scientific consensus regarding the most effective treatment strategy. Cell transplantation methods vary widely among the trials conducted in cell source, dose, transplantation route, and transplantation timing [43,44,45]. Therefore, there is a need for generating better evidence on the effects of MSC therapy in SCI. Herein, we present 17 clinical studies that used MSCs as a therapeutic approach for the treatment of SCI, focusing on critical analysis with an emphasis on their design and quality.

## 2. Materials and Methods

### 2.1. Study Design and Data Search Strategies

**Figure 1:**
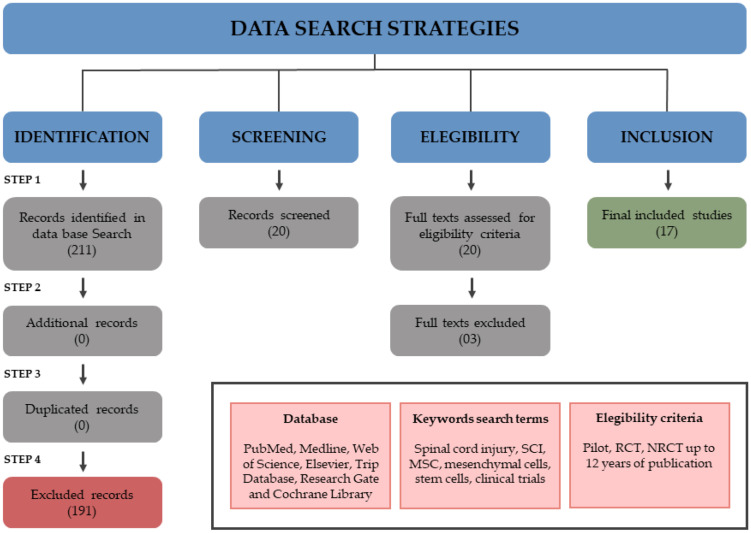
Data search strategies for clinical trials about MSCs in SCI.

This is a literature review of clinical trials in MSC therapy for SCI. Literature searches in the following databases were performed to identify relevant studies: PubMed, Medline, Web of Science, Elsevier, Trip Database Research Gate, and Cochrane Library. Keyword search terms included spinal cord injury, SCI, MSC, mesenchymal cells, stem cells, clinical trials. Studies from up to 12 years ago at publication were included. Languages were restricted to Portuguese, Spanish, and English. The data search strategy is shown in Figure 1.

### 2.2. Selection Criteria

Pilot studies, non-randomized clinical trials, and randomized clinical trials were included. Reviews, case reports, case series, and pre-clinical studies were excluded. Only studies where clinical intervention was limited to administration of MSCs to adult participants (minimum 5 participants per study) with neurological impairments caused by SCI were eligible for this review.

### 2.3. Data Extraction

A total of four reviewers evaluated the full texts from the screened articles, and those studies that did not meet the inclusion criteria were excluded. The remaining studies were read fully and included or excluded based on the eligibility criteria. In order to search for studies that by chance may not have been found in the initial search, the references of each included study were searched again for additional findings. Nineteen references were found from 210 records, and the 191 excluded did not match the eligibility criteria. Three more studies were excluded because of the following reasons: classified as case reports (N = 2) and no study protocol description (N = 1). At the end of the search, 17 clinical trials were included in this review. A summary of the studies reviewed is presented in Figure 1.

Information gathered from the selected trials included: study design, sample size, characteristics of lesion, cell source, follow up, adverse events, tools for evaluating motor and sensory gains, clinically significant outcomes, and other details that could influence the quality of a clinical trial.

## 3. Results and Discussion

### 3.1. Clinical Trials’ Main Variables 

Table 1 depicts some variables identified in the 17 studies selected which are relevant to clinical trials using stem cells for spinal cord injury. These variables are: stem cell therapeutic schema, cell sources, routes of administration, SCI level and ASIA grade, phases of spinal injury, associated interventions, evaluation of functional and motor/sensory improvements, and main outcomes. 

### 3.2. The Importance of Grade, Level, and Severity of a Lesion

Spinal cord lesions have several relevant factors besides trauma kinematics, such as the heterogeneous nature of such injuries (grade, level, and severity), and the time elapsed between the occurrence of the trauma and the establishment of a potentially effective treatment. Considering the degree of SCI, the American Spinal Injury Association (ASIA) impairment scale (AIS) defines the issue as follows: A = Complete: no sensory or motor function is preserved in the sacral segments S4–S5. B = Incomplete: sensory but not motor function is preserved below the neurological level and includes the sacral segments S4 and S5. C = Incomplete: motor function is preserved below the neurological level, and more than half of key muscles below the neurological level have a muscle strength grade <3. D = Incomplete: motor function is preserved below the neurological level, and at least half of key muscles below the neurological level have a muscle strength grade greater than or equal to 3. E = Normal: sensory and motor function are normal [46]. 

A recent systematic review and meta-analysis including 114 studies with poor quality overall evaluated variables with prognostic significance for neurological outcomes after early decompression surgery. Severity, level and mechanism of injury, and type and time of treatment were analyzed. Neurological recovery was significantly different between all grades of SCI severity, in the following ASIA grade order: C > B > D > A [16]. Functional neurological recovery was associated with the level of injury, being higher in the lumbar spine, intermediate in the cervical and thoracolumbar spine, and lower in thoracic spine. A retrospective and longitudinal analysis reported improved conversion rates in low thoracic (T10–T12) SCI injuries when compared with high/mid thoracic (T2–T9) SCI injuries [47]. Therefore, lesion characteristics may influence the functional recovery after stem cell transplantation.

The trials analyzed here included various types of SCI functional characteristics, classified according to grade, level, and severity (Table 1). Considering ASIA grade, nine of them (53%) included specifically subjects classified as ASIA A; two other studies (11.7%) enrolled subjects with ASIA A and B; one study (5.9%) restricted the inclusion to ASIA B subjects; another study (5.9%) showed more flexibility, including ASIA B, C, and D, but excluded ASIA A classification; and the last four clinical studies included all ASIA grades (23.5%). Regarding the level of lesion, three studies (17.6%) included only subjects with cervical lesions; eight studies (47%) had cervical and thoracolumbar lesions; four of them (23.5%) included thoracolumbar; and two (11.7%) included only subjects with thoracic-level injuries. Therefore, the lesion profile varied widely among the 17 studies, and together with other factors may have influenced the effects of the MSC transplantation in the clinical trials evaluated (Figure 4). In fact, one study evaluated possible correlations between lesion characteristics and motor/sensory gains, as an exploratory analysis [48].

### 3.3. Spinal Cord Injury Phases

The phase of the SCI may exert an important influence on the results in a study of cell therapy. As described in Table 1, the majority of studies included subjects in a chronic phase (11 studies, 64.7%), and four studies (23.5%) had a sub-acute phase in their eligibility criteria. Only two studies (11.7%) included subjects in the acute phase. Some findings in pre-clinical studies support the choice of subacute and chronic lesions: neuronal regrowth can occur in long-standing injuries, and it seems to be greater in delayed transplantation compared to early-phase transplantation [49]. Another experimental study shows that increased TGF-1 and attenuation of pro-inflammatory cytokine gene expression in a non-acute phase of SCI might create a more favorable environment for transplanted cells and promotion of axonal regrowth [50]. El-Kheir (2014), in a phase I/II controlled single-blind clinical trial using autologous adherent bone marrow cells in SCI subjects who were included in a chronic phase, suggested that cell transplantation in a microenvironment with a diminished inflammatory response, which is seen in the chronic and perhaps in subacute phases, may have a greater probability of success [51].

### 3.4. Adverse Events (AE)

**Figure 2:**
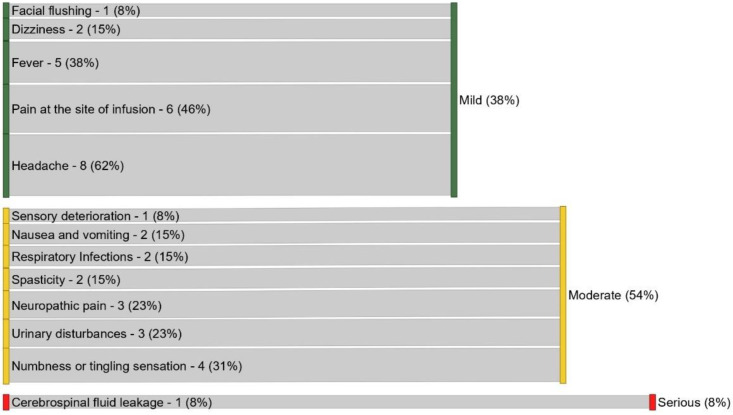
Intensity of adverse events reported (n = 13) in 17 clinical studies.

Careful analysis of the adverse events is important to demonstrate the safety of MSC transplantation in the SCI clinical setting. Various AE (n = 13) were reported in the 17 reviewed studies, as shown in Table 2, and the rate by intensity is represented in Figure 2. Among the 13 events reported, the most commonly mentioned event was headache (n = 8; 62%), followed by pain at the site of lesion (n = 6; 46%). Regarding the intensity of these AE, the majority of them were considered moderate, with simple resolution after 48–72 h post-injection, and in some cases, use of medication was necessary. Only one study reported a serious adverse event (liquoric fistula) that was corrected by a surgical procedure. The proportion of studies with no adverse events reported was 17.6% (n = 3). Overall, the analysis demonstrated promising safety data for the transplantation of MSCs via different routes of administration, and different phases and levels of lesion, although restricted only to the limited periods of follow-up performed in the reviewed trials.

### 3.5. Sample Size

**Figure 3:**
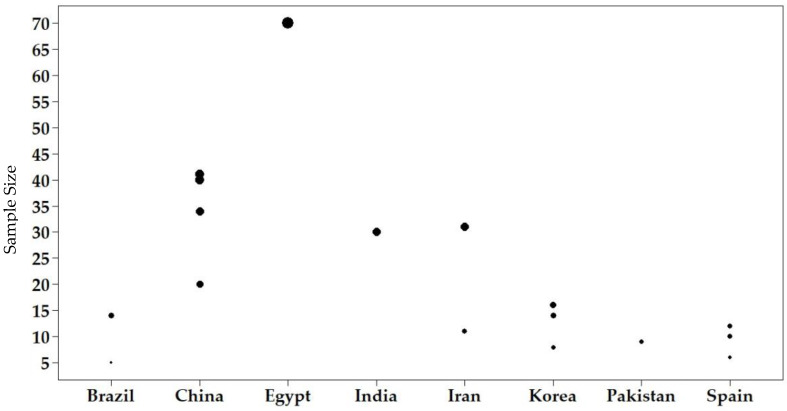
Sample size and country distribution of clinical studies reviewed.

A significant sample size is necessary to validate studies developed with humans and to make randomization possible. However, trials in stem cell therapy frequently do not include a suitable number of participants. Many variables may contribute to an inadequate number, such as a small target population that meets the necessary methodological recommendations and need to establish homogeneous groups, in order to avoid allocation bias and tendency results. Figure 3 shows the heterogeneity of sample sizes in the 17 reviewed studies, varying from minimum 5 to 70 subjects each, although most studies included a small number of participants. Pilot studies may have small samples, but it is expected that in phase 2/3 clinical studies, a significant number of subjects will be used, in order to be representative within a specific population.

### 3.6. Cell Sources 

**Figure 4:**
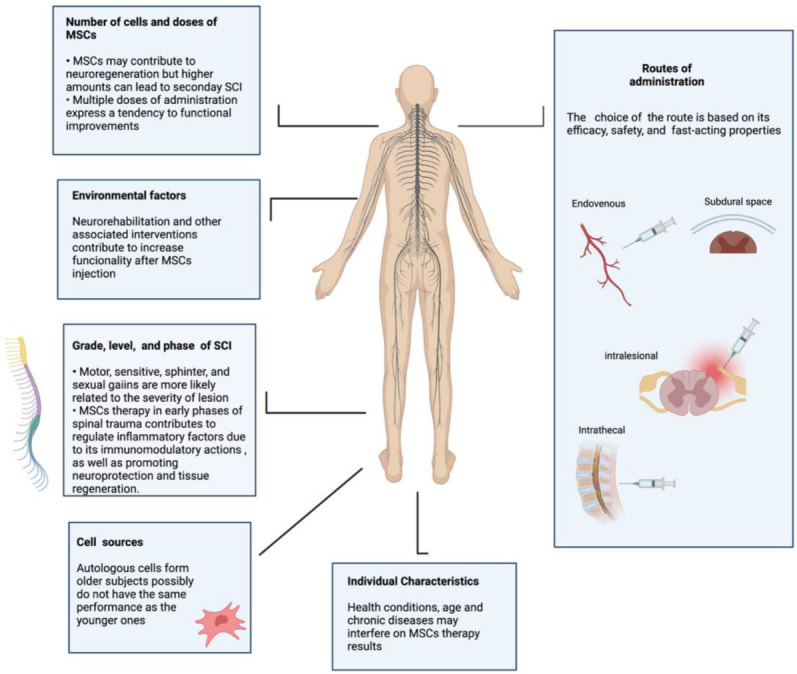
Potential factors influencing MSCs’ efficacy in SCI.

MSCs have been the most studied stem cell type for treating spinal injuries [52,53]. However, currently, we cannot confirm if there is a better MSC source for the treatment of spinal cord injuries. The studies here reviewed adopted the following cell sources: autologous MSCs derived from bone marrow (n = 12, 70%), bone marrow MSCs combined with Schwann cells (n = 1, 5.8%), autologous adipose tissue MSCs (n = 2, 11.7%), and allogeneic MSCs derived from umbilical cords (n = 1, 5.9%), as described in Table 1. Regarding the origin of cells for transplant, most of selected clinical studies had autologous cells as the first choice (n = 15, 88.2%) and two studies used allogeneic cells (n = 2, 11.7%). Autologous MSCs have several advantages, such as lack of post-transplant rejection, no requirement for cell line development, and low start-up costs, among others [5]. On the other hand, the lack of standard markers, differences in laboratory procedures, type and age of the source tissue, and individual health conditions (Figure 4) may affect the purity of MSCs pool and impair their effectiveness for clinical applications, making possible the occurrence of poorer performance than allogeneic MSCs [54]. A large cell production can be achieved using allogeneic cell sources, which can be used for many patients in an efficient way. Other advantages can be identified too, such as permanent cell availability, quality control (QC) that can be applied to larger lots, emergency indications being expected, and no patient biopsy being needed [55,56]. Due to the fact that only two studies using allogeneic cell sources were evaluated in our review, more data are needed from allogeneic MSC transplant patients to draw conclusions about this methodology.

### 3.7. Routes of Administration 

Different pathways of stem cell infusion are available, as shown in Figure 4, and therefore advantages and disadvantages must be considered when conducting a clinical study for a certain disease or condition, such as SCI [57]. Table 1 shows that 9 of the 17 studies (53%) we reviewed used the intrathecal route of administration, whereas the others used intralesional, intravenous, subarachnoid, and subdural routes. The intrathecal route was shown to be safe for MSC injection in SCI patients with various lesion characteristics. Among the various cell therapy routes tested for spinal cord injury, the intrathecal injection has the advantage of being easy for multiple injections and may represent a relatively direct and fast-acting route [44,51,58,59,60,61,62,63,64]. Furthermore, it is suggested that local injections of MSCs may be more effective than systemic approaches [65,66].

### 3.8. Therapeutic Scheme

Studies involving MSCs as therapeutic source for spinal cord lesions have to compete to be the best therapeutic scheme, considering cell number, doses administered, and therapeutic effect. A high concentration of cells should be produced in a small volume, in order to avoid secondary damage to the spinal cord due to the volume injected. Although previous trials suggested that multiple administrations of cell therapy could promote better recovery than a single MSCs application [56,60,67], this issue still needs to be clarified. The cell therapy products described in the reviewed studies did not follow any methodological pattern for application, and they did not allow the determination of optimal conditions for dosage, number, and interval of stem cell injection. However, despite the heterogeneity, clinical improvements were reported (Table 1), although there is still a need for confirmation of these beneficial effects.

### 3.9. Follow-up

The monitoring of short, middle, and long-term adverse events, and possible benefits related to an intervention, can be registered in a well conducted follow-up in clinical trials. Small follow-up trials may fail to identify and measure adverse events, mainly those that appear in the mid-term and long-term, but also the rare ones [68,69,70]. Moreover, monitoring the evolution of function in its motor, sensory, and cognitive aspects is necessary, as it is one of the main parameters showing the beneficial effects of cell therapy. 

Although some studies suggest functional gains mainly in the first 90 days after transplantation [48], it is understood that other findings during and after this period can be considered clinically significant. Moreover, and if sustained rehabilitation and effort are associated, functional improvement can continue over one year or more years [51,71,72], and recovery rates were positively correlated with longer follow-up duration [43,73]. 

The studies analyzed here described different follow-up times of participants submitted to stem cells transplantation (Figure 7). The heterogeneity of follow-ups of the studies reviewed varied from 1 to 36 months, as follows: six studies (35.2%) had a 6-month period, three studies (17.6%) had a 12-month follow-up, and seven studies (41%) had different periods of follow up. All these variabilities make it difficult to register evidence of unexpected and long-term adverse events, and to parameterize aspects related to the neuroresponsiveness of the nervous system to MSC transplantation. Thus, in this scenario, it is reasonable to state that MSC transplantation is a safe procedure, at least in the time frames evaluated in the studies reviewed here, in a population of 371 participants altogether.

### 3.10. Associated Interventions

Although rehabilitation alone has not been shown to promote significant improvements in patients with complete spinal cord injury, it offers constant stimuli for relearning and brain readjustment through neuroplasticity, creating efficient mechanisms for the execution of movements. In addition, it is plausible to consider that exercising paralyzed limbs may have a positive effect on the restoration of neurological function [74,75]. 

The use of rehabilitation with MSC transplantation was not included in all the articles reviewed: only 5 of 17 studies performed this combined therapeutic approach (Table 1). Even though the methodologies of rehabilitation varied among them, regarding the period of physical therapy treatment (ranging from weeks to months), the frequency of exercise performance (ranging from 1 to 5 days a week), and the duration of the daily treatment (ranging from minutes to hours per day), the five studies that used rehabilitation included global exercises and specific training for bladder and bowel function, respiration, and assisted movement of the upper and lower limbs. 

Some considerations about the issues here discussed may influence the results of MSC therapy in spinal injury and are presented in Figure 4.

### 3.11. Outcomes 

**Figure 5:**
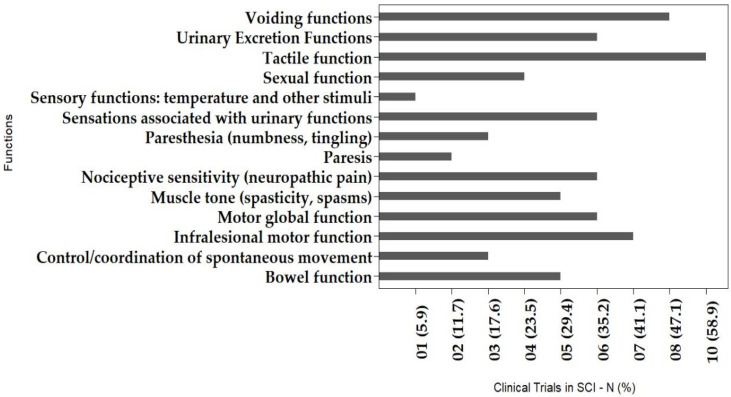
Clinically significant outcomes observed after MSC therapy in SCI, considering the domains of body structures and functions.

**Figure 6:**
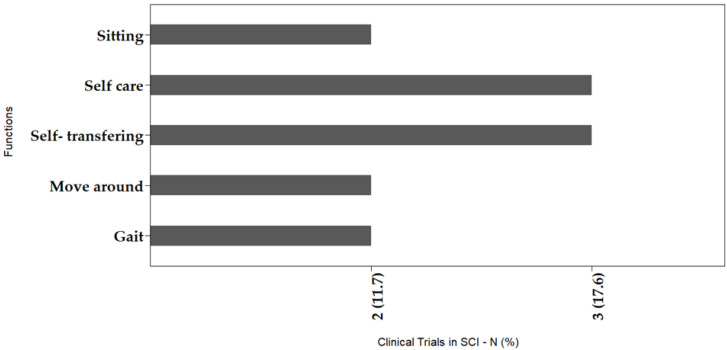
Clinically significant outcomes after MSC therapy in SCI, considering the domains activity, participation, and performance.

Overall, the identified improvements described in the articles reviewed are listed in Table 1 and graphically represented in Figure 5 and Figure 6. Regarding the domains of body structures and functions (Figure 5), 14 outcomes were reported. Eight (57.1%) of the total are related to sphincter controls and the sensory system; one outcome (7.1%) is related to control and coordination; one outcome (7.1%) is related to muscle tone; another outcome was sexual function; and three outcomes (21.4%) is related to the motor system. These gains may be associated with improvements in quality of life, although no article here reviewed had reported the use of any tool to measure this variable. However, regarding specifically movement and functional improvements, even though they were less frequently described, there was a trend towards greater improvements in those studies that combined MSC transplantation with a rehabilitation program. This may corroborate the data by Satti et al. (2016), which indicated a positive effect for combining cell therapy with rehabilitation resources, as it complements the trophic and therapeutic effects of transplanted stem cells [59].

Motor, sensory, and functional improvements showed in Figure 5 and Figure 6 are clinically significant, and are conceptualized according to the instrument recommended by the World Health Organization for Disabilities, the International Classification of Functioning, Disability and Health (ICF). Figure 5 presents the main gains observed, considering the structures and body functions, showing the connections to the organs or body segments which had improvements after MSC transplantation. In Figure 6 the gains are presented according to human activities and participation, where self-care and transferring were the most commonly identified improvements, followed by sitting, moving around, and gait, the last of which was the outcome least reported among the studies. We consider this classification as a marker of the beneficial impact that the MSCs had in subjects with SCI, and thereafter, in their quality of life, regardless of statistically significant results. 

### 3.12. Methods for Evaluating Gains in Motor/Sensory Impairments and Human Functioning

Improvements in sensory and motor functions have been reported in most clinical studies with transplanted MSCs in situations of traumatic spinal cord injury. The reviewed studies employed different instruments for measuring functioning and sensory-motor responses to identify improvements. The ASIA scale has been universally applied by researchers to generate evidence of therapeutic effects, and in the 17 reviewed studies it was employed as the primary outcome measurement, although the data were presented in different ways (averages, absolute values, and percentages) [15,44,48,51,58,59,60,61,62,63,64,67,76,77,78,79]. For the analysis of the global functioning, changes according to the ASIA scale have been widely considered a reliable strategy, and it is strongly recommended. However, there are gains in human functioning that cover different domains, including motor, sensory, and sexual aspects, which influence labor and daily living activities. Consequently, they influence the subject’s ability to perform these tasks, showing improvements in the global context of the handicaps caused by the spinal cord injuries. Other tools for measuring functional gains were reported in the reviewed studies and are briefly described below: specific neurological scales, neurophysiological tests, spasticity scales, sensory tests, urodynamic tests, pain scales, sphincter function scales, and image exams. 

Spasms and spasticity were evaluated in the analyzed studies through the Ashworth and Penn scales, and two studies showed reductions in these characteristics, improving muscle function [56,60]. One study described an increase in spasticity, so it was reported as an adverse event (Table 2), although the results were not statistically significant. 

Urodynamic studies were reported in most of the reviewed clinical trials, but information about the level and quality of improvements is not well defined [48,60,61,62,78,79]. Urinary control as an outcome of the recovery of bladder function had promising results in early phases [77]. For chronic neurogenic bladder assessment, possible beneficial effects were reported through the Geffner scale. To evaluate bowel dysfunction, the most cited tool was the Neurogenic Bowel Dysfunction (NBD) scale, which was reported by all reviewed studies.

Neurophysiological studies showed improvements in the somatosensory evoked potential, with response to sensory and motor nerve conduction suggesting a trend and improvement in these functions by MSC therapy [51,60,61,67,76,79]. Surface electromyographic parameters have been used to estimate activity patterns of muscles that act in normal and pathological human gait, and in other movements and activities involving voluntary motricity differentiating distinct electrical activity in muscles during the performance of tasks of varying difficulty [80,81], where such electrical signals evidence active muscle reinnervation [60,61,62]. 

Serial magnetic resonance imaging (MRI) changes were also found after MSC application in subjects with spinal injuries, such as enlargement of the spinal cord, decreases in cavity size, and the appearance of fiber-like, low-signal-intensity streaks [71,82]. In this review, structural changes in MRI were identified in two clinical studies, which reported an improvement in the syrinx size [62] and blurring of the cavity margin, along with the appearance of dark fiber-like streaks [67].

Quality of life (QOL) was not assessed in any of the 17 studies. Since the WHO considers QOL a health condition parameter, we consider it important for such studies, although it is a subjective parameter. Moreover, evidence-based medicine actually includes patients’ experiences and preferences as one of the pillars for its consolidation and development.

Three studies had randomized designs including controls and intervention groups [51,77,78]. They applied different methodologies, varying in source of cells, therapeutic scheme, route, level of spinal cord injury, and grade of lesion. The sample sizes varied to (20, 15, and 10 subjects in the intervention groups), and the adverse events reported were mostly considered mild, though one was classified as moderate. Follow-up periods varied from 6 to 18 months. This information shows heterogeneity in the data, making a comparison among them difficult. However, statistically significant improvements were reported by the three studies, in both sensitive and motor ASIA scores, mainly in functioning and sphincter activities [51,77,78], and one of the three also mentioned changes in ASIA grade [77]. 

### 3.13. Quality in Clinical Trials

SCI clinical trials need to be well designed and conducted carefully and safely to demonstrate the necessary evidence for the use of MSCs. They must consider and document both benefits related to the treatment, and the predictable and unpredictable risks. Several methodologies contribute to assessing the quality of evidence, in addition to the design of a study. The level of evidence represents our confidence that the effect estimate is adequate to support a decision or recommendation. Considering the need for clinical trials of good quality, it is necessary to have high methodological rigor for a lower risk of bias. 

A large number of studies have provided important insights into the safety and feasibility of stem cell transplantation. There is a need, however, for larger randomized trials to demonstrate the efficacy of MSC therapy. In order to avoid allocation biases and trends, randomization is mandatory in clinical studies to guarantee that groups keep balanced as much as possible. Proposed by Hill in 1948, randomized clinical trials (RCT) represent, among a wide universe of study designs, the gold standard parameters for assessing therapeutic interventions [83,84]. 

There are other variables beyond randomization that help improve the quality of a clinical study, such as blindness and matching. If sample is mostly homogeneous considering the features among subjects, one must decrease the possibility that more bias will arise in the study. Having total blinding and matching in a unique clinical study reinforces that the only differences between the groups will be the experimental and control interventions. Since these are the only differences between the groups, if changes occur in the outcomes at the end of the study, then these findings can be attributed to the intervention (transplanted MSCs). Important concepts in quality that may influence the efficacy and effectiveness of a study, impacting the power, reliability, and generation of evidence, which need to be suitable enough to support a decision or recommendation, should be considered. 

**Figure 7:**
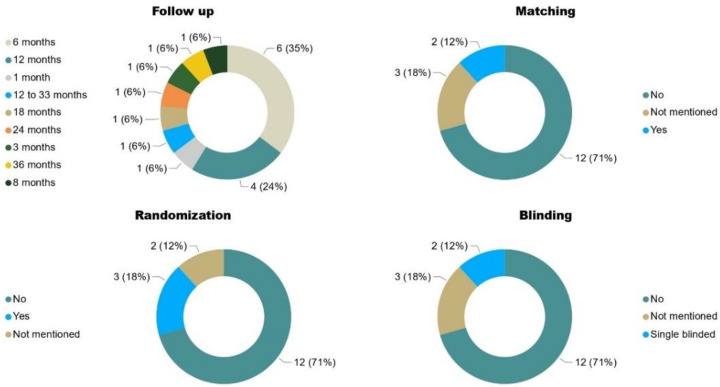
Quality criteria for conducting clinical trials: follow-up, matching, randomization, and blinding.

**Figure 8:**
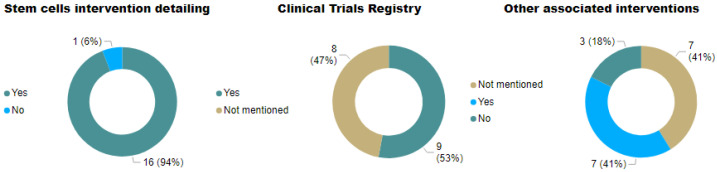
Quality criteria for conducting clinical trials: MSCs details, associated interventions, and CT registry.

The variables from the 17 studies are listed in Table 3 and graphically represented in Figure 7 and Figure 8, and all of them show high heterogeneity. Figure 7 shows the variables follow-up time, matching, randomization, and blinding. The variable follow-up, described in both months and percentages, was already discussed in Section 3.9. Among the studies reviewed, 35.2% (6 studies) performed a 6 month follow-up; 17.6% (3 studies) performed a 12 month follow-up, and only 6% (1 study) had a follow-up after 12 months. Regardi9ng matching the samples included, only 11.7% (2 studies) presented this information, whereas all the others were classified as “not mentioned” or “no matching”.

Randomization was performed in 17.6% (3) of the 17 studies reviewed, 11.7% (2) did not mention whether subjects were randomized, and 64.7% did not randomize their participants. Considering blinding, no studies were fully blinded; 11.7% (2) were single-blinded, 17.6% (3) did not mention blinding, and 64.7% (11) were unblinded. Figure 8 shows the details of the cell therapy approaches, other associated interventions, and registry in the clinical trials. Most of the studies presented the necessary details of the cell therapy approach (88.2%, n = 15). Additionally, 41% (7) included rehabilitation, but two of them were established as standard treatments; 17.6% (3) did not mention it; and 35.2% (6) had no other intervention associated with MSC transplantation. About registration in clinicaltrials.gov, 53% (9) stated that they registered the study, whereas 41% (7) did not mention it. 

All variables mentioned above are strongly recommended in clinical trials using MSCs, and the absence of some of these tools will weaken the assessment and reduce the strength of the study. 

### 3.14. Quality Control in Stem Cell Manufacturing

The International Society for Stem Cell Research (ISSCR) recommends that universal standards must be established to enable comparisons of cellular identity, purity, and potency, which are critical when comparing studies; to ensure reliability of the dose–response ratio; and to assess mechanisms of toxicity [85]. However, even though there are guidelines available to support the use of MSCs, such as Food and Drug Administration (FDA) and European Medicines Agency (EMA) guidelines, there is no standardized definition of quality control tests and methodologies that should be applied in cell therapy using MSCs.

The official regulatory documents concerning advanced medicinal products for human use with a specific focus on cell therapy from competent authorities, such as European (EMA), American (FDA), and Brazilian (ANVISA) regulatory agencies, recommend that the product’s qualification by the manufacturer must follow some requirements. The specifications for release testing are product-specific and include identity, purity, potency, impurities, sterility, potency, cell viability, and total cell number, which are considered essential features for the therapeutic use of MSCs [86,87,88]. 

The recognition of a human cell as an MSC follows some minimal criteria defined by The International Society for Cellular Therapy (ISCT). These requirements are plastic adherence in standard culture conditions; a certain phenotypic profile (expression of CD105, CD73, and CD90; and lack of expression of CD45, CD34, CD14 or CD11b, CD79alpha or CD19, and HLA-DR surface molecules); and the ability to differentiate into osteoblasts, adipocytes, and chondroblasts in vitro [89]. 

**Figure 9:**
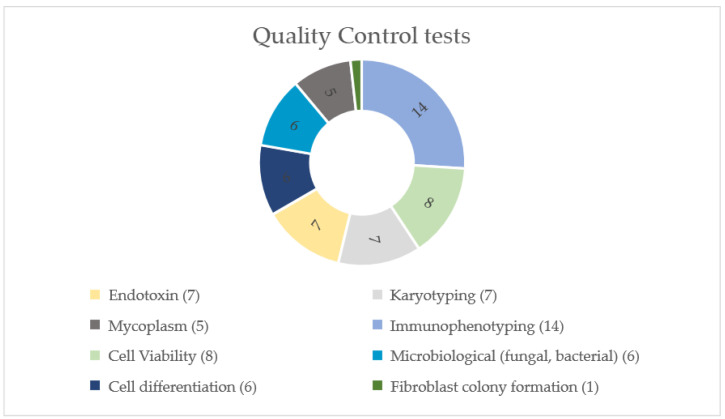
Types of quality control tests performed during stem cell manufacturing in the reviewed clinical trials on SCI.

**Figure 10:**
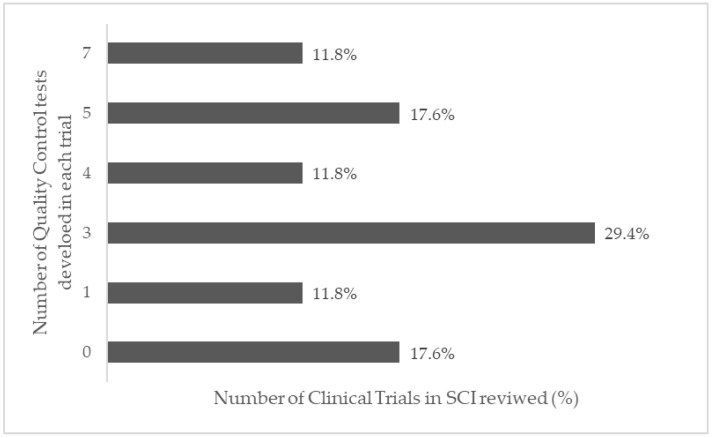
Number of quality control tests performed in the clinical trials on SCI.

The main quality control tests performed in the reviewed clinical trials are shown in Figure 9: 14 studies performed immunophenotyping tests (82.4%); 8 studies performed cell viability (47.1%); 7 performed endotoxin tests (41.2%); 7 performed karyotyping (41.2%); 6 performed fungal and bacterial microbiological analysis (35.3%); 6 performed cell differentiation (35.3%); 5 performed mycoplasm tests (29.4%); and only 1 performed a fibroblast colony formation test (5.9%). Considering the 14 studies that mentioned quality controls tests, the distribution among them varied between 1 and 7 (Figure 10). Three studies (17.6%) did not mention quality control tests.

### 3.15. Ethical Aspects

The use of stem cells for therapeutic purposes needs to consider ethical and regulatory aspects in order to guarantee the development of clinical trials in good clinical practice worldwide. For the development of advanced medicinal products in Europe and the United States, and some Asian countries, there is a regulatory framework that is well defined by the regulatory agencies. However, most South American countries do not have their own regulations, with the exception of Brazil. In this review, all 17 studies were previously submitted to local or national ethics committees and collected informed consent signatures from all subjects enrolled, following ethical guideline provisions from the Helsinki Declaration. Six of them (35%) were submitted for regulatory agency approval (three to the Korean Ministry of Food and Drug Safety and three to the Spanish Agency for Medicaments and Health Products). Brazil had only recently established its regulatory rules, and thus the two publications could not be submitted to the regulatory agency at that period. In addition to the information made available by the regulatory agencies, such as the FDA and EMEA, international societies such as International Society of Stem Cell Research (ISSCR) and International Society of Cell and Gene Therapy (ISCT) provide useful guidelines for conducting clinical studies following ethical and good clinical practices for cell therapy development [85,86,87,88,89]. 

## 4. Conclusions

**Figure 11:**
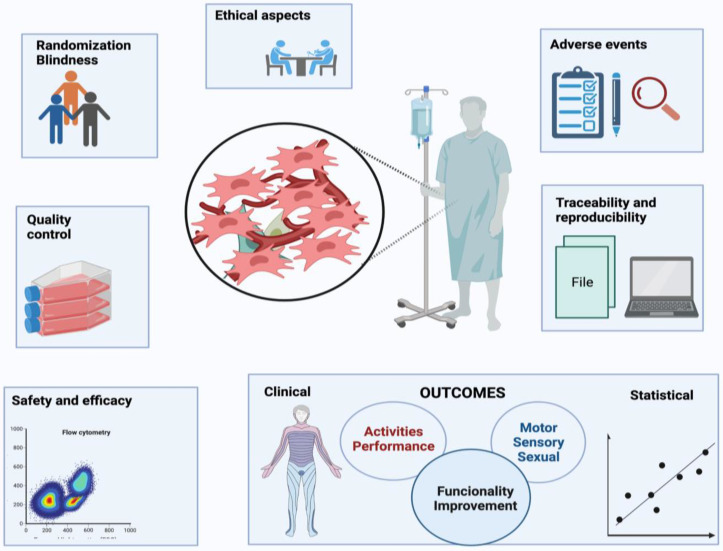
General qualifiers for MSC clinical trials on SCI: a summary of important points of care: methodology, ethics, good clinical and manufacturing practices and outcomes. Each variable can contribute to the performing of a good quality trial using MSCs for spinal injuries.

**Figure 12:**
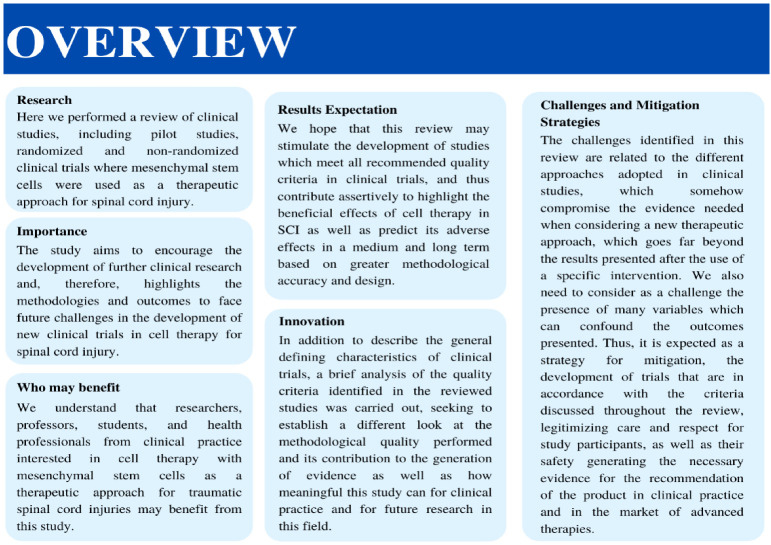
Overview of the main contributions of this article to perform good quality clinical trials in SCI: research, importance, benefits, results expectation, innovation, challenges and mitigation strategies.

After analyzing the results shown in the selected studies, we found that the therapeutic use of MSCs is a safe treatment for people with SCI in different phases and conditions, at least in the short term. Adverse events were in the majority of studies mild, and no long term EAS was registered. Additionally, it was noticed that clinically and statistically significant gains were presented, strengthening and ratifying the potential beneficial effects of mesenchymal cells on SCI, and hence, on the patients’ quality of life. In this scenario, motor and sensory improvements were classified as structural, that is, in relation to organic functions and systems. The functioning gains were associated to patients’ activities, performance, and social participation in the most diverse situations of daily life. However, due to the expressive methodological heterogeneity of the clinical studies and the small samples enrolled, in addition to methodological issues that reduced the quality of the clinical trials, we suspect confounding variables. Moreover, a long-term follow-up is needed to determine the durability of the gains observed. Some important qualifiers in this context are shown in Figure 11. A continuous search to find the best cell source and transplantation protocol should proceed, in order to consolidate the use of MSC therapy in SCI. Large trials are necessary, following all the methodological parameters of quality mentioned here, using standard evaluations, such as the ASIA scale, and other variables such as neurological recovery and quality of life. A summary of important contributions addressed in this review are presented on Figure 12.

## Figures and Tables

**Table 1:** Main variables described in the reviewed clinical trials.

Cell Therapy Approach	Cell Source	Routes of Administration	SCI Level/ASIA Grade	SCI Phase	Interventions Associated	Tools for Evaluating Functioning and Motor/Sensory Improvements	Clinically Significant Outcomes	Statistically Significant Outcomes	References
1 × 10^6^ cells/kg	Autologous BM	Intrathecal	Cervical and Thoracic AIS A	Sub-acute, Chronic	No	ASIA, BI, SSEP, MEP, NCV, MRI	Improvement in bladder function, supportive walking and sitting	No	Pal et al., 2009
4 × 10^8^	Autologous AD	Intravenous	Cervical and thoracolumbar AIS A, B	Chronic	No	ASIA, SCIM, VAS, MRI, MEP, SEP	improvement in Self-care	SEP in 3 subjects and ASIA A to C in 1 subject	Ra et al., 2011
7 × 10^5^ to 1.2 × 10^6^	Autologous BM	Intrathecal	Thoracic AIS A	Acute and Sub acute	Rehabilitation	ASIA	5 Subjects changed from AIS A to C	No	Karamouzian et al., 2012
25 μL–8 × 10^5^ cells/μL	Autologous BM	Intralesional	Cervical AIS A	Chronic	No	ASIA, RUV, EMG, PSSEP, MRI	Improvement in ASIA score, residual urine volume	ASIA score, residual urine volume	Dai et al., 2013
1 × 10^8^ cells in 5 mL	Autologous BM	Intralesional	Cervical and thoraco lumbar AIS A-, B, and C	Acute, Sub acute and Chronic	No	ASIA, BI, ASHWORT	Benefits in AIS grading and score, bowel, and urinary function, reduction of pain, erectile dysfunction and hypertonia	Not mentioned	Jiang et al., 2013
2 × 10^6^ cells/kg 1–8 monthly injections	Autologous-BM	Intrathecal	Cervical and thoracic AIS A and B	Chronic	Rehabilitation	ASIA, SSEP MRI, FIM	Improvement of neurological and functional measures	Motor and sensory improvement	El-Kheir et al., 2014
2 doses (50 µL to 4 × 10^5^ cells/μL)	Allogenic UC	Intralesional	Thoraco Lumbar AIS A	Chronic	Neurological rehabilitation	ASIA, BI, MMS, MTS	Significant and stable improvement in movement, self-care ability, and muscular tension; residue urine volume	Strength of waist, abdomen, and lower limbs increased, excessive muscle tension decreased, and self-care ability	Cheng et al., 2014
5 × 10^6^ cells/cm^3^	Autologous BM	Intralesional	Thoraco Lumbar AIS A	Chronic	Rehabilitation	ASIS, SSEP, MRI, VAS, USD	Improvement in urologic function, lower limb sensitivity and motor function, reduction in pain	ASIA sensitivity and motor scores	Mendonça et al., 2014
2 × 10^7^ cells	Autologous BM	Intralesional	Thoraco Lumbar AIS A	Chronic	No	ASIA, SSEP, MRI, SCIM, FIM, USD	Improvement in sexual dysfunction, urinary bladder-filling sensation and sphincter control.	Bowel regularity	Larocca et al., 2016
2 or 3 injections (1.2 × 10^6^/kg)	Autologous BM	Intrathecal	Thoracic AIS A	Sub-acute, Chronic	No	ASIA, MRI	Not mentioned	Not mentioned	Satti et al., 2016
3–7 injections; 100 × 10^6^ to 230 × 10^6^ cells. additional dose (30 × 10^6^ cells) after 3 months	Autologous BM	Intrathecal	Thoracic AIS A	Chronic	Not mentioned	ASIA, FIM, IANR-SCIFRS, BI, Ashworth, Geffner, VAS, MRI, USD	Improvement in sensitivity and sphincter (urinary and bowel) control, infralesional motor activity, decreases in spasms and spasticity, improvement in sexual function	Sphincter control, reduction of neuropathic pain and spasticity	Vaquero, 2016
4 doses 3 × 10^7^ cells day 1, 4, 7 and 10 months (120 × 10^6^ total)	Autologous BM	Intrathecal	Cervical and thoraco Lumbar AIS B, C or D	Chronic	Not mentioned	ASIA, FIM, IANR-SCIFRS, BI, Ashworth, Geffner, VAS, MRI, USD	Sensitivity and motor improvement, reduction of pain	Sensitivity and motor improvement	Vaquero et al., 2017
3 doses 100 × 10^6^ (1, 4 e 7 months)	Autologous BM	Intrathecal	Cervical, thoracic and lumbar AIS A, B, C and D	Chronic	Not mentioned	ASIA, FIM, IANR- SCIFRS, BI, Ashworth, Geffner, VAS, MRI, USD	Improvement in sensitivity, in neurogenic bowel and bladder dysfunction, spasms and spasticity, neuropathic pain, and voluntary muscle contraction	Pin prick score assessment, improvements in the syrinx size	Vaquero et al., 2018
(9 × 10^7^ céls)	Autologous AD	Intrathecal	Cervical and thoraco Lumbar AIS A, B, D	Sub-acute, Chronic	Not mentioned	ASIA, MRI, EMG, SSEP, MEP	Improvement in ASIA motor score, Voluntary anal contraction improvement	Not mentioned	Hur et al., 2016
2 doses: 1.6 × 10^7^ + 3.2 × 10^7^	Autologous BM	Intralesional/subdural	Cervical AIS B	Chronic	Rehabilitation	MEP, SEP, MRI	Improvement in motor grade of the upper extremities and ADL; Increases in spinal cord diameter; disappearance of the cavity; SEP and MEP improvements	Not mentioned	Oh et al., 2016
5 × 10^7^	Autologous BM and SC	Intrathecal	Cervical, thoracic and lumbar AIS A	Subacute	Rehabilitation	ASIA, SCIM-III, EMG, NCV, MRI, UDS	Recovery of trunk movement and equilibrium in standing/sitting positions, reduction in the severity of constipation, sensation of the filling bladder, voiding.	Score Sensory (pinprick and light touch); SCIM III total score respiration and sphincter management, mobility, and self-care;	Oraee-Yazdani 2021
5 × 10^7^/kg	Allogenic UC	Subarachnoid	Cervical, thoracic, thoracolumbar ASIA A-D	Chronic		ASIA, IANR-SCIFRS, MRI Penn scale, Geffner, Neurogenic Bowel Dysfunction score, RUV	Improvements of pinprick, light touch, motor and sphincter scores. Decrease in muscle spasticity	Improvements in muscle spasm, autonomic system, bladder and bowel functions, RUV and MRI	Yang et al., 2020

AD = adipose; ASIA = American Spinal Injury Association; AIS = America International Spinal Injury Scale; BI = Barthel’s index; BM = bone marrow; EMG = electromyography; FIM = functional independence measure; IANR-SCIFRS = International Association of Neurorestoratology—Spinal Cord Injury Functional Rating Scale; MEP = motor-evoked potentials; MRI = magnetic resonance imaging; MMS = manual muscle strength; MTS = muscle tension scale; NVC = nerve conduction velocity; PSSEP = paraspinal somatosensory evoked potential; RUV = residual urine volume; SCIM = spinal cord independence measure; SSEP = Somato-sensory evoked potentials; UC = umbilical cord; UDS = urodynamic study; VAS = Visual Analog Scale; SC = Schwann cells.

**Table 2:** Adverse events reported in reviewed clinical trials.

References	Fever	Numbness or Tingling	Facial Flushing	Headache	Neuropathic Pain	Spasticity	Pain at the Site	Dizziness	Cerebrospinal Fluid Leakage	Respiratory Infections	Nausea and Vomiting	Sensory Deterioration	Urinary Disturbances
Pal et al., 2009	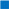	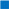	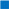	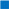	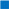	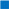	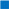	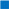	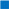	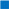	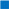	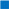	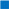
Ra et al., 2011	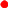	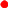	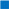	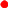	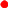	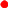	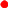	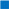	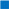	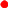	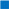	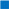	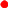
Karamouzian et al., 2012	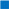	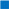	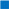	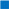	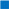	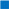	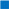	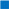	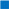	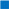	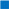	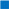	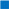
Dai et al., 2013	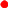	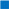	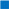	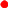	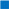	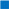	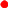	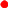	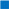	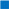	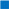	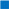	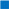
Jiang et al., 2013	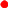	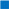	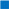	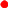	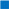	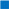	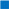	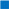	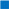	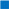	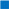	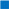	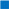
El-Kheir et al., 2014	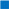	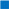	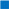	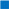	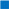	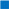	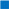	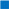	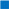	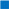	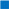	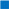	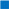
Cheng et al., 2014	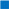	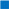	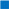	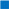	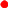	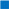	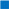	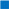	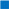	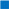	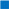	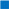	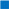
Mendonça et al., 2014	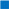	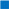	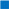	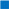	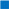	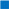	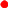	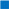	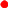	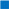	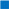	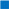	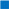
Larocca et al., 2017	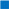	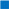	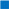	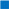	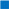	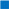	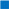	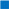	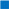	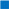	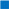	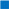	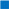
Satti et al., 2016	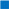	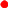	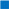	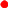	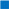	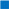	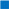	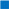	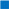	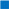	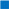	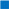	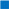
Vaquero et al., 2016	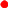	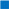	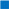	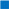	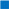	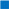	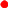	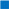	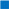	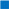	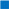	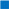	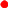
Vaquero et al., 2017	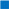	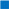	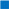	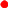	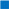	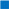	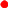	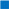	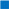	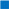	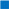	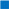	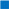
Vaquero et al., 2018	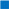	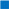	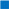	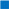	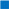	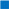	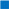	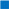	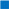	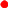	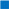	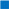	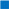
Hur et al., 2016	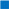	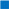	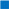	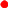	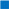	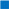	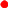	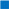	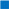	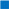	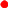	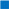	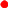
Oh et al., 2016	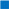	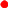	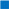	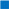	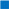	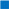	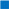	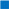	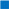	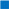	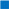	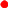	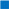
Oraee-Yazdani et al., 2021	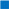	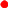	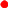	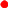	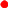	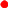	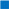	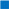	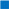	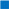	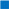	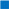	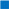
Yang et al., 2021	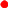	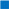	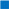	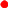	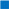	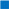	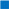	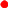	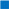	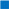	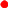	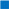	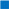

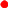
 AE present; 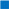 AE absent.

**Table 3:** Quality criteria SCI clinical trials.

References	Clinical Trial Phase	Description of Study Design	Criteria of Eligibility Well Defined	Cell Therapy Intervention Detailing	Other Associated Interventions	Group Control	Sample Size	Randomization	Allocation Sequence Method	Blinding	Matching	Description of Detailed Clinical Features	Clinical Trials.Gov Registry
El-Kheir et al., 2014	Phase 1/Phase 2	Yes	Yes	Yes	Yes	Yes	70	Yes	Not mentioned	Single blinded	Not mentioned	Yes	Yes
Pal et al., 2009	Pilot clinical study	Yes	Yes	Yes	Not mentioned	No	30	Not mentioned	Not mentioned	Not mentioned	Not mentioned	No	Yes
Ra et al., 2011	Phase 1	Yes	Yes	Yes	Not mentioned	No	8	Not mentioned	Not mentioned	Not mentioned	Not mentioned	Yes	Yes
Oh et al., 2016	Phase 3	Yes	Yes	Yes	Yes	No	16	No	No	No	No	Yes	Not mentioned
Hur et al., 2016	Pilot clinical study	Yes	Yes	Yes	No	No	14	No	No	No	No	Yes	Not mentioned
Karamouzian et al., 2012	Phase 1/Phase 2	Yes	Yes	Yes	Yes	Yes	31	No	No	No	No	Yes	Not mentioned
Dai et al., 2013	Not mentioned	Yes	Yes	Yes	Yes	Yes	40	Yes	No	Single blinded	Yes	Yes	Not mentioned
Cheng et al., 2014	Phase 2	Yes	Yes	Yes	No	Yes	34	Yes	Not mentioned	Not mentioned	Yes	Yes	Yes
Jiang et al., 2013	Not mentioned	Yes	Yes	Yes	No	No	20	No	No	No	No	Yes	Not mentioned
Mendonça et al., 2014	Phase 1	Yes	Yes	Yes	Yes	No	14	No	No	No	No	Yes	Yes
Larocca et al., 2016	Phase 1	Yes	Yes	Yes	Not mentioned	No	5	No	No	No	No	Yes	Yes
Satti et al., 2016	Phase 1	Yes	Yes	Yes	Yes	Yes	9	No	No	No	No	Yes	Yes
Vaquero et al., 2016	Phase 1/Phase 2	Yes	Yes	Yes	Not mentioned	No	12	No	No	No	No	Yes	Yes
Vaquero et al., 2017	Phase 2	Yes	Yes	No	Not mentioned	No	10	No	No	No	No	Yes	Yes
Vaquero et al., 2018	Phase 2	Yes	Yes	Yes	Not mentioned	No	6	No	No	No	No	Yes	Not mentioned
Oraee-Yazdani et al., 2021	Phase 1/Phase 2	Yes	Yes	Yes	Yes	No	11	No	No	No	No	Yes	Not mentioned
Yang et al., 2021	Phase 1/Phase 2	Yes	Yes	Yes	Not mentioned	No	41	No	No mentioned	No	No	Yes	Not mentioned

## Data Availability

Datasets were generated during the study. We endorse MDPI Research Data Policies.
